# Ordinal reduction of frontotemporal sulcal asymmetry with increasing auditory verbal hallucinations in schizophrenia spectrum disorders

**DOI:** 10.1038/s41537-026-00821-3

**Published:** 2026-10-23

**Authors:** Shun-Chin Jim Wu, Héloïse de Vareilles, Pilar Salgado-Pineda, Lucila Barbosa, Joan Soler-Vidal, Paola Fuentes-Claramonte, Pablo del Olmo-Encabo, Emili Inarejos-Clemente, Begoña Hoyos, Núria Ramiro, Jesús Ruiz, Fernando Boatas, John Suckling, Edith Pomarol-Clotet, Peter J. McKenna, Graham K. Murray

**Affiliations:** 1 Department of Psychiatry, University of Cambridge, Cambridge, UK; 2 FIDMAG - Germanes Hospitalàries Research Foundation / CIBERSAM-ISCIII, Barcelona, Spain; 3 Hospital Nostra Senyora de Meritxell, Servei Andorrà d’Atenció Sanitària, Escaldes-Engordany, Andorra; 4 Departament de Psicologia Bàsica, Clínica i Psicobiologia, Universitat Jaume I, Castelló, Spain; 5 Radiology Department Sant Joan de Déu, Barcelona, Spain; 6 Fundació Hospitalàries Sant Boi, Barcelona, Spain; 7 Fundació Hospitalàries Barcelona, Barcelona, Spain; 8 Fundació Hospitalàries Barcelona Nord, Barcelona, Spain; 9 Fundació Hospitalàries Martorell, Barcelona, Spain; 10 Cambridgeshire and Peterborough NHS Trust, Cambridgeshire, UK

**Keywords:** Biomarkers, Psychosis

## Abstract

Asymmetry in paracingulate sulcus (PCS) length and superior temporal sulcus (STS) depth was examined in schizophrenia spectrum patients stratified by auditory verbal hallucination (AVH) frequency. An ordinal reduction in sulcal asymmetry was observed with increasing AVH frequency, reaching statistical significance only for STS depth. The decline in effect size from frequent to less frequent AVH may explain prior inconsistent findings from binary AVH classifications that disregard clinical heterogeneity.

## Introduction

Leftward asymmetry of the paracingulate sulcus (PCS) length and rightward asymmetry of superior temporal sulcus (STS) depth are more pronounced in humans than in other great apes^1–5^. The STS is a primary sulcus within the auditory cortex that develops early in gestation, remains stable into adulthood, and shows relatively high heritability, particularly for sulcal depth^2,6,7^. In contrast, the PCS is a tertiary sulcus in the medial frontal region that develops later in gestation, becomes prominent during adolescence, and shows lower heritability overall, with sulcal length being least genetically determined, and more strongly influenced by prenatal environmental factors^1,7–9^. Reductions in these human-enhanced asymmetries have been reported in schizophrenia spectrum disorders and linked to auditory verbal hallucinations (AVH)^10,11^, a predominantly human symptom, suggesting that STS and PCS asymmetry may serve as complementary neurodevelopmental markers bridging the bimodal (early- vs. late-developing) sulcal model and the lateralization theory of psychosis^6,12^.

However, significant findings across studies in schizophrenia remain inconsistent, with studies having negative or equivocal findings for STS depth and for PCS length asymmetry. It is possible that some of this heterogeneity reflects the use of binary definitions of AVH^10,11,13,14^. Such classification may conceal clinically meaningful findings, as AVH vary in frequency and severity; for example, one study found that only around 44% of patients with schizophrenia experience frequent hallucinations^15^. Accordingly, pooling frequent and less frequent AVH within binary categories may dilute true effect.

To address this limitation, we extended our previous work on binary AVH classifications and frontotemporal sulcal asymmetry in psychosis^10,11^ by examining an independent cohort of patients with schizophrenia spectrum disorders stratified by AVH frequency, including continuous/near-continuous AVH, weekly to daily AVH, and no lifetime experience of AVH. We tested whether PCS length and STS depth asymmetry showed an ordinal reduction with increasing AVH frequency, and quantified effect sizes to assess how clinical heterogeneity influenced the detectability of asymmetry alterations.

## Materials and methods

Structural T1-weighted MRI data were acquired from 33 patients with continuous/near-continuous AVH (Constant AVH), 28 patients experienced weekly to daily AVH (Weekly+ AVH), 31 patients without lifetime AVH (Never AVH), and 58 healthy controls (total N = 150). AVH frequency was assessed using item 1 of the Psychotic Symptom Rating Scale for Auditory Hallucinations^16^: Constant AVH scored 4, Weekly+ AVH scored 1-3, and Never AVH scored 0 with absence of lifetime AVH confirmed through clinical record review and interviews with both the patient and their treating psychiatrists. All the patients met DSM-5 criteria for schizophrenia or schizoaffective disorder based on structured clinical interview^17^, and all participants were right-handed. Healthy controls were recruited from the same geographic area and matched to patients for age and sex. Exclusion criteria for all participants included age <18 or >65 years, history of neurological disorder or traumatic brain injury, and alcohol or substance abuse within 12 months prior to participation. Patients with estimated premorbid IQ < 70, assessed using the Word Accentuation Test (Test de Acentuación de Palabras, TAP)^18^, were excluded. Healthy controls with a personal or family history of psychiatric illness or a personal history psychotropic medication were also excluded based on the Spanish version of the Structured Clinical Interview for DSM-5 (SCID-5). Demographic and clinical characteristics are presented in Table 1.

**Table 1:** Demographics and clinical information.

Demographic and clinical profiles						
	Constant AVH	Weekly + AVH	Never AVH	Healthy Controls	Test statistic	*p*-val
**Sample size**	33	28	31	58		
**Age (mean, SD)**	40.3 (10.4)	42.4 (11.3)	45.1 (10.1)	39.7 (12.5)	F _(3,146)_ = 1.71	0.17
**Onset age (mean, SD)**	23.5 (7.4)	25.2 (8.8)	26.9 (10.3)	n/a	F _(2, 85)_ = 1.06	0.35
**Male (%)**	24 (73%)	18 (64%)	20 (65%)	35 (60%)	χ2 (3) = 1.41	0.70
**Schizophrenia (%)**	29 (88%)	28 (100%)	31 (100%)	n/a		0.03^**+**^
**Premorbid IQ (mean, SD)**	98.5 (7.5)	98.3 (11.1)	104.4 (9.3)	102.4 (8.1)	F _(3,144)_ = 3.63	0.01
**IQ (mean, SD)**	90 (23.3)	91.2 (16.7)	100.2 (18)	105 (14.2)	F _(3,140)_ = 6.43	<0.001
**PANSS P3 (median, IQR)**	5 (1)	4 (2)	1 (0)	n/a	H (2) = 69.3	<0.001
**PANSS Positive (median, IQR)**	16 (6)	15.5 (4.5)	10 (5)	n/a	H (2) = 1.49	0.48
**PSYRATS AVH (median, IQR)**	29 (8)	20 (12.3)	0 (0)	n/a	H (2) = 68.3	<0.001
**PSYRATS Total (median, IQR)**	40 (19)	31.5 (16.3)	0 (8.5)	n/a	H (2) = 34.7	<0.001
**Total antipsychotics Chlorpromazine equivalent dose (mean, SD)**	560 (318)	550 (348)	371 (259)	n/a	F _(2,76)_ = 3.14	0.05

Constant AVH = patients with continuous or near-continuous auditory verbal hallucinations per day, Weekly+ AVH = patients with at least one auditory verbal hallucinations per week but not near-continuous, Never AVH = patients without any auditory verbal hallucinations, SD = standard deviation, IQR = Interquartile range, PANSS P3 = Positive and Negative Syndrome Scale-Hallucinatory Behaviour Item, PSYRATS AVH= Psychotic Symptom Rating Scales-Auditory Hallucinations Subscale, F = one-way ANOVA, χ^2^ = Chi-squared test, ^**+**^ = Fisher’s Exact Test, H = Kruskal Wallis test.

All participants underwent high-resolution T1-weighted MRI on the same 3 T Philips Ingenia scanner at Sant Joan de Déu Hospital, Barcelona, between July 2017 and July 2024 using a standardized protocol (matrix 320 × 320 × 250; voxel size 0.75 × 0.75 × 0.8 mm³; TR = 8.4 ms; TE = 3.8 ms). No major protocol or software changes occurred during data acquisition. Images were processed using the Morphologist pipeline in BrainVISA in March 2025, including tissue segmentation, surface reconstruction, and cortical fold graph generation. The PCS was manually labelled on unlabeled cortical fold graphs following a previously validated protocol^19^, blind to diagnosis and contralateral sulcal patterns, while the STS was automatically labelled using BrainVISA’s sulcal recognition pipeline (version 5.0.4)^20^. PCS length, STS mean depth, estimated intracranial volume, and hemispheric brain volumes were automatically extracted in December 2025. All labels and metrics were visually inspected for quality and outliers by two raters (HdV & SCJW) blinded to clinical data.

Sulcal asymmetry was quantified using an asymmetry difference (Left - Right). Between-group differences were tested using one-way analysis of covariance (ANCOVA) with 5000 permutations (Manly procedure), controlling for age, sex, estimated intracranial volume, hemispheric brain volume difference, and the sum of bilateral metrics. Significant omnibus effects were followed by parametric family-wise error rate (FWER)-controlled post hoc Tukey honest significant difference tests. Effect sizes were estimated using Partial Eta-Squared (η^2^_p_) for omnibus tests and Hedges’ g with small-sample correction for post hoc Tukey tests. Homogeneity of variance was tested using the Bartlett test and influential observations were evaluated using Cook’s distance. Independence between the two target variables (PCS length and STS depth) was evaluated using Spearman correlation.

Additional models included premorbid IQ (TAP), PANSS positive score minus item P3 (hallucinatory behaviors), and antipsychotic dose (chlorpromazine equivalents), and unilateral metrics were examined as sensitivity analyses. An additional permutation ANCOVA (5000 permutations) tested the ordinal relationship between AVH frequency and sulcal asymmetry in patients with schizophrenia spectrum disorders, with AVH frequency coded as Never = 1, Weekly + = 2, and Constant = 3. Within-group asymmetry was assessed secondarily using two-sided one-sample Wilcoxon signed-rank tests, with effect sizes estimated as matched-pairs rank-biserial correlations (r). All of the above analyses were conducted using the R statistical software (version 4.2.3), along with the following packages: stats (version 3.6.2), rstatix (version 0.7.2), lmPerm (version 2.1.0), multcomp (version 1.4-25) and ppcor (version 1.1).

### Ethics

All participants gave written informed consent prior to participation in accordance with the Declaration of Helsinki. This study was approved by the Clinical Research Ethics Committee of Fidmag Germanes Hospitalaries [PR-2021-15 and PR-2018-27].

## Results

**Fig. 1:**
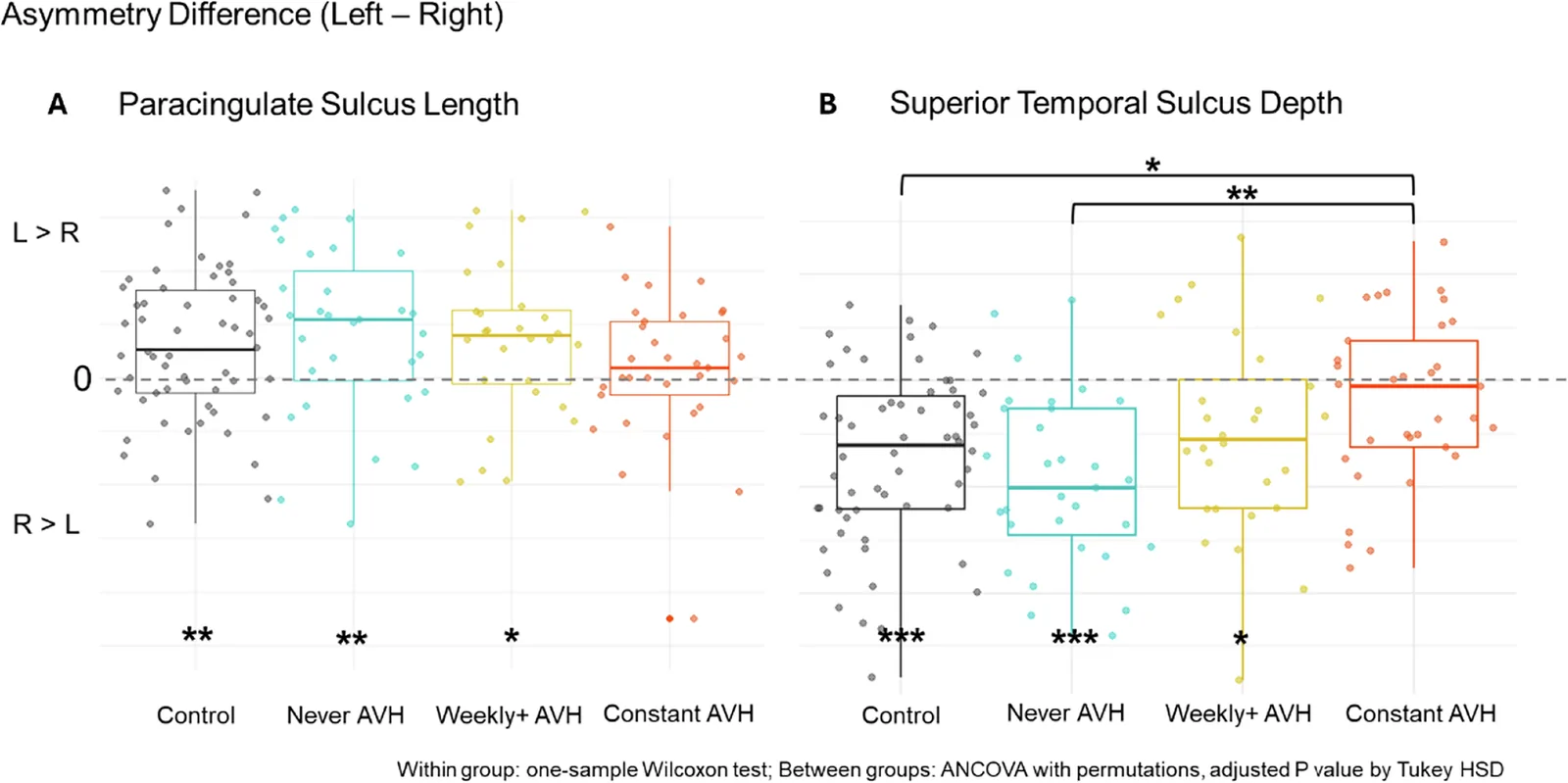
Reduced asymmetry of the paracingulate sulcus length and superior temporal sulcus depth in patients with constant auditory verbal hallucinations. **A** Patients with constant auditory verbal hallucinations (Constant AVH) showed a loss of leftward asymmetry in paracingulate sulcus length (*P* = 0.21), assessed by the asymmetry difference (left – right; L - R) using a one-sample nonparametric Wilcoxon signed-rank test against zero. In contrast, patients experiencing hallucinations at least weekly (Weekly+ AVH) showed reduced asymmetry, while patients without auditory verbal hallucinations (Never AVH) and healthy controls exhibited more pronounced or preserved leftward asymmetry. However, between-group differences were not statistically significant with one-way analysis of variance (ANOVA) with 5000 permutations. **B** Constant AVH patients also demonstrated a loss of rightward asymmetry in superior temporal sulcus depth (*P* = 0.30), assessed using a one-sample Wilcoxon test. Weekly+ AVH patients showed reduced asymmetry, whereas Never AVH patients and healthy controls showed more pronounced or preserved rightward asymmetry. Notably, Constant AVH patients exhibited significantly reduced rightward asymmetry compared with Weekly+ AVH, Never AVH, and control groups (one-way permutation ANCOVA, 5000 permutations; F(3,141) = 4.45, η^2^_p_ = 0.09, *P* = 0.003). Post hoc tests revealed mean asymmetry differences of 1.48 mm (SE = 0.43, *P* = 0.003) and 1.09 mm (SE = 0.39, *P* = 0.03) between Constant AVH and Never AVH/controls, respectively. *Asterisks denote statistical significance from one-sample Wilcoxon tests (below) and post hoc Tukey HSD tests following permutation ANCOVA (above): *P < 0.05, **P ≤ 0.01, **P ≤ 0.001. ANCOVA covariates included age, sex, estimated total intracranial volume, interhemispheric brain volume difference (left – right brain volumes), and the sum of bilateral metrics (left + right length/depth). Positive values (L > R) indicate that the left sulcal metric is greater than the right, whereas negative values (R > L) indicate that the right metric is greater than the left. Boxplots show the median, interquartile range (IQR), and whiskers extending to 1.5 × IQR; individual data points are overlaid.

The omnibus ANCOVA revealed a significant group effect for STS depth asymmetry (F(3,141) = 4.45, η^2^_p_ = 0.09, *P* = 0.003), but not for PCS length asymmetry (Fig. 1). Post hoc analyses revealed patients with Constant AVH exhibited significantly reduced rightward STS depth asymmetry compared with Never AVH patients (mean difference = 1.48 mm, SE = 0.43, Hedges’ g = 0.88, *P* = 0.003) and healthy controls (1.09 mm, SE = 0.39, Hedges’ g = 0.64, *P* = 0.03). Homogeneity of variance was satisfied, and no influential outliers were found for either metric. PCS length and STS depth were not correlated (rho = -0.02, *P* = 0.77). Group means, standard deviations, and effect sizes are provided in Table S1.

Sensitivity analyses confirmed that reduced STS depth asymmetry in Constant AVH patients remained significant after adjusting for premorbid IQ, PANSS positive score minus P3, and antipsychotic dose. The ordinal trend analysis showed a significant association between increasing AVH frequency and reduced STS depth asymmetry (F(1,85) = 9.21, *P* = 0.003), but not PCS length asymmetry (*P* = 0.125). Unilateral analyses likewise showed significantly reduced right STS depth in Constant AVH patients compared with Never AVH patients and healthy controls (all *P* ≤ 0.004; Table S1). Within-group analyses demonstrated that only patients with Constant AVH showed no significant asymmetry in either PCS length or STS depth, as asymmetry differences did not differ significantly from zero (PCS: r = 0.22, *P* = 0.21; STS: r = 0.18, *P* = 0.30). Patients with Weekly+ AVH exhibited significantly reduced asymmetry on both metrics (PCS: r = 0.44, *P* = 0.02; STS: r = 0.47, *P* = 0.01), whereas healthy controls and Never AVH patients showed preserved or slightly increased asymmetry (controls: PCS r = 0.39, *P* = 0.003; STS r = 0.70, *P* < 0.001; Never AVH: PCS r = 0.49, *P* = 0.005; STS r = 0.70, *P* < 0.001; Fig. 1).

## Discussion

This study extends the previous findings of reduced frontotemporal sulcal asymmetry in patients with schizophrenia spectrum disorders^10,11^ by demonstrating, in an independent cohort, that STS depth showed a significant group-level ordinal relationship with AVH frequency, while PCS length appeared to exhibit a similar ordinal trend without reaching statistical significance. The STS findings remained robust after adjustment for additional covariates. These findings suggest that PCS and STS asymmetry are not merely binary markers of lifetime AVH but show an ordinal reduction with increasing AVH frequency. Consistent with these between-group findings, secondary within-group analyses showed that only the Constant AVH group lacked significant asymmetry in PCS length and STS depth, whereas Weekly+ AVH showed intermediate reductions and Never AVH preserved or slightly increased asymmetry. Furthermore, the group-level findings partially align with prior hypotheses that reduced PCS length and STS depth asymmetry, reflecting bimodal sulcal deviations at different developmental stages^6^.

The effect was most robust for STS depth, a genetically conserved metric^6^, potentially implicating genetically influenced human-enhanced sulcal features in AVH-related neuroanatomy. A key advance is that AVH frequency substantially influences effect size estimates: larger asymmetry difference was observed between Constant AVH and Never AVH than between Weekly+ AVH and Never AVH, indicating that clinically meaningful subgroups can be obscured in binary classifications. Previous studies likely vary in the proportion of patients with continuous AVH in their samples, and do not always specify this; no previous study of PCS and STS metrics has examined this group separately, offering a possible explanation for the inconsistently significant findings of prior studies that relied on binary AVH classifications. For example, a recent study examining the PCS length asymmetry reported an effect size of g = -0.27 for lifetime AVH versus Never AVH^10^. Such an effect size could potentially arise from a simulated weighted combination of a large effect size (0.41) in a subgroup with continuous AVH (44%) in schizophrenia^15^ and a smaller effect size (0.08) in a group with less frequent AVH (assumed 56%) (-0.23 = -0.41 × 0.44 + -0.08 × 0.56). These results suggest that in the future, detailed AVH phenotyping could improve the detectability of AVH-related neuroanatomical effects.

## Supplementary information

Table S1

## Data Availability

The dataset analysed during this study is available from the FIDMAG Research Foundation on reasonable request.

## Materials availability

Correspondence regarding data curation, imaging and statistical analyses, and manuscript content should be addressed to Shun-Chin Jim Wu and Graham K. Murray. Requests for data access should be directed to Pilar Salgado-Pineda, Edith Pomarol-Clotet, and Peter J. McKenna.
