# Supplementary material for: Ordinal reduction of frontotemporal sulcal asymmetry with increasing auditory verbal hallucinations in schizophrenia spectrum disorders
Source: Schizophrenia (Heidelb). 2026 Oct 23;12(1):88. doi: 10.1038/s41537-026-00821-3 (PMC13754816; doi:10.1038/s41537-026-00821-3)
Supplement: Supplementary file 1 [file 41537_2026_821_MOESM1_ESM.docx]

**Supplementary Materials**

**Supplementary Results**

Table S1. Paracingulate and Superior Temporal Sulcus Metrics, Asymmetry, and between-group comparisons (ANCOVA)

**Table S1. Paracingulate (PCS) and Superior Temporal Sulcus (STS) Metrics, Asymmetry, Sum (mean and standard deviation in mm), and between-group comparisons (ANCOVA)**

|  | **Constant AVH** | **Weekly+**  **AVH** | **Never**  **AVH** | **Healthy Controls** | **F_;_η^2^_p_** | **p-val** | **g1** | **g2** | **g3** | **g4** | **g5** | **g6** |
| --- | --- | --- | --- | --- | --- | --- | --- | --- | --- | --- | --- | --- |
| **Left PCS length** | 30.8  (23.5) | 42.6  (27.1) | 44.9  (30.0) | 43.2  (28.3) | 1.66;  0.03 | 0.18 | -0.51 | -0.10 | -0.4 | 0.02 | -0.41 | 0.12 |
| **Right PCS length** | 27  (31.1) | 25.2  (21.9) | 24.2  (27.5) | 28  (25.6) | 0.04;  <0.01 | 0.99 | 0.07 | 0.00 | 0.03 | -0.04 | 0.07 | -0.04 |
| **PCS length AD** | 3.9  (34.3) | 17.4  (35.8) | 20.8  (38.9) | 15.2  (35.6) | 1.03;  0.02 | 0.37 | -0.41 | -0.08 | -0.30 | -0.04 | -0.37 | 0.11 |
| **PCS length**  **sum** | 59.7  (42.9) | 67.8  (33.8) | 69.1  (42.3) | 71.2  (40.6) | 0.57;  0.01 | 0.54 | -0.32 | -0.07 | -0.26 | -0.02 | -0.25 | 0.06 |
|  | **Constant AVH** | **Weekly+**  **AVH** | **Never**  **AVH** | **Healthy Controls** | **F_;_η^2^_p_** | **p-val** | **g1** | **g2** | **g3** | **g4** | **g5** | **g6** |
| **Left STS depth** | 13.7  (2.6) | 13.9  (1.6) | 13.6  (1.2) | 14.2  (1.8) | 0.67;  0.01 | 0.57 | 0.08 | 0.15 | -0.22 | -0.14 | -0.08 | -0.30 |
| **Right STS depth** | 14.1  (1.51) | 14.9  (1.63) | 15.5  (1.63) | 15.6  (1.53) | 6.62;  0.12 | <0.001 | -0.88  ** | -0.39 | -0.92  *** | -0.43 | -0.49 | -0.04 |
| **STS depth AD** | -0.40  (1.58) | -1.03  (1.89) | -1.88  (1.61) | -1.46  (1.63) | 4.45;  0.09 | 0.004 | 0.88  ** | 0.50 | 0.64  * | 0.27 | 0.37 | - 0.23 |
| **STS depth sum** | 27.7  (2.6) | 28.7  (2.6) | 29.1  (2.3) | 29.8  (2.9) | 3.23; 0.06 | 0.03 | -0.48 | -0.14 | -0.68  * | -0.34 | -0.33 | -0.20 |

Constant AVH = patients with continuous or near-continuous auditory verbal hallucinations per day, Weekly+ AVH = patients with at least one auditory verbal hallucination per week but not near-continuous, Never AVH = patients without any auditory verbal hallucinations, AD = asymmetry difference (Left - Right), F = One-way ANCOVA with 5000 permutations, η^2^_p_ = Partial Eta-Squared, g1 = Hedges’ g, Constant AVH vs. Never AVH; g2 = Weekly+ AVH vs. Never AVH; g3 = Constant AVH vs. healthy controls; g4 = Weekly+ AVH vs. healthy controls; g5 = Constant AVH vs. Weekly+ AVH; g6 = Never AVH vs. healthy controls. *Asterisks denote statistical significance from family-wise error rate (FWER)-controlled post hoc Tukey HSD tests following one-way analysis of variance (ANOVA) with 5000 permutations (**P* < 0.05, ***P* ≤ 0.01, ***P* ≤ 0.001). Covariates in ANCOVA include sex, age, and estimated total intracranial volume for the unilateral metrics and sums. Additional covariates for asymmetry differences including the interhemispheric brain volume difference (left - right brain volumes), and the sum of bilateral metrics (left + right length/depth).
